# Longitudinal small and medium enterprise (SME) data on survival, research and development (R&D) investment, and patent applications in Korea's innovation clusters from 2008 to 2014

**DOI:** 10.1016/j.dib.2019.103967

**Published:** 2019-05-22

**Authors:** Byung-Keun Kim, JungTae Hwang

**Affiliations:** a School of Industrial Management, Korea University of Technology and Education, Cheonan, Chungcheongnam-do, Republic of Korea; b Department of Business Administration, Hallym University, Chuncheon, Gangwon-do, Republic of Korea

**Keywords:** Firm survival, Innovation cluster, Longitudinal data, Recession period

## Abstract

This article contains survey data from 588 firms on 1) their length of survival, 2) technological innovation related information, such as research and development (R&D) investment, research manpower, and the number of patent applications, along with 3) other basic data on firm size and affiliated industry sector. The dataset was extracted from firms residing in three different innovation cluster regions of Korea. All the data in this article are based on firm level questionnaire in the innovation cluster regions, with the exception of the firm survival information extracted from the National Tax Service of Korea and industry information from “Statistics Korea”. The related research article using the current dataset was published under the following title: “Does R&D investment increase SME survival during a recession?” Jung et al., 2018.

**Table:** Specifications table

Subject area	*Management, Business*
Subject area subdivision	*Innovation Management*
Type of data	*CSV files, SPSS script file, and figures, tables in the article.*
How the data was acquired	*Special request to access the proprietary database built on annual survey of Korea's innovation clusters.* National Tax Service compiled in Korea Enterprise Data (CRETOP^®^).
Data format	*Raw (but extracted from the fore-mentioned database, based on firm size and age), the original raw database file is not open to public.*
Experimental factors	*The firm sample was filtered using data older than three years of age and with multiple responses.* *The dataset was combined with firm survival information.*
Experimental features	*Panel data on firm attributes (related to technological innovation) and firm output, survival, and sales.* *Basic survival analysis*
Data source location	*Three innovation clusters in South Korea (Daejeon, Daegu, Gwangju)*
Data accessibility	*Data included in this article.*
Related research article	*A relevant research article to this dataset is* H. Jung, J. Hwang, B. Kim, Does R&D investment increase SME survival during a recession?, Technol. Forecast. Soc. 137, 2018, 190–198 [1]*.*

**Table:** 

**Value of the data** • The dataset presents rare information on the lifespan of 588 firms over 72 months from 2008 to 2014, including basic information on firm size measured in terms of sales and employees. This dataset is useful for the survival analysis of small and medium enterprises whose information is not publicly available. • The dataset contains longitudinal firm level data of small and medium enterprises (SMEs). Most of this information is related to innovation such as patent applications, annual R&D investment (total R&D and internal R&D use), and annual R&D manpower. Consequently, the dataset is particularly useful for those who study innovative SMEs and performance of SMEs. • The information on innovative venture business certificate issued by Korean government and information on export status also provides valuable opportunities to study both traditional vs. innovative SMEs and domestic vs. non-domestic SMEs. • The data could be useful if future research investigates regional innovation clusters as it contains the regional code of three innovation clusters.

## 1 Data

The cross-sectional dataset consists of 588 firms that were filtered as participating in multiple surveys during 2008–2014. Firms with fewer than 500 employees are included in the dataset. Even though the data collection in Korea's innovation clusters began in 2006, the current dataset in this data article only contains collections from 2008, which is due to the associated research [1] demanding the dataset after the 2008 financial crises. The dataset is valuable since it is hard to obtain innovation related information and business closure information together on small and medium enterprises (SMEs). In the case of small firms, researchers can obtain such information only if they are public firms listed on stock market. Each firm's data contains the descriptive variables shown in Table 1. Table 2 describes the variables that have postfix year-name variables. Each variable in Table 2 has a series of seven variables; for example, Sales 09 means the total firm sales of 2009. The variable constitutes a series from Sales 08 to Sales 14. The dataset of the first supplementary file, described in Table 2, is a specific form of “wide-file” for executing SPSS cox regression with time-varying covariates. According to Guo [2], SPSS and SAS require “wide-file” format where time-varying covariates are organized by several variables. However, STATA requires “long-file” format where each subject occupies more than one data line for varying covariates. For the purpose of sharing dataset with wider academic communities, we provide a separate supplementary file with “year” as an independent variable for those who prefer standardized panel data format (and for STATA users) without complex postfix year name. These twin supplementary files constitute main dataset files of this article. In addition, a separate supplementary file (the third supplementary file) on industry concentration and industry growth during the period is also presented in this data article, and is illustrated in Table 3. Finally, the fourth supplementary file contains exemplary SPSS processing algorithm for survival analysis.

**Table 1:** Description of firm attributes.

Variables	Data Type	Content
Month_Time	Integer	Life span as number of months from Dec. 2008, 72 means right censored (survival until Dec. 2014)
Event	Binary	0 means closure of business, 1 survival
Founding_year	Integer	Founding year of the firm (at least before 2006)
KSIC_	2 digit Integer	Similar to Standard Industry Code (Korean version revision KSIC 2007 [3])
Region	Code (1/2/3)	1 Daeduk, 2 Gwangju, 3 Daegu

**Table 2:** Description of attributes of firms that have annual values.

Variables	Data Type	Content
Number_EmployeeXX	Count	Number of firm employees in year 20XX:2008–2014
SalesXX	Integer	Total sales in year 20XX (Unit: million KRW)
Res_Dev_InvestXX	Integer	Total R&D investment in year 20XX
Internal_RnD_InvestXX	Integer	Internal R&D investment in year 20XX
ResearchPersonnelXX	Count	Number of researchers in year 20XX
Patent_ApplicationXX	Count	Number of domestic and international patent applications in year 20XX
VentureCertXX	Binary	Venture certificate (annual renewal, government acknowledgement of innovative firms) in year 20XX
ExportDumXX	Binary	Existence of export sales in year 20XX
CRXX	%	Firm's associated industry concentration in terms of the top three firms' market share in year 20XX
GRXX	ratio	The growth of the firm's affiliated industry in year 20XX (increment of industry production over the production of the previous year)

**Table 3:** Description of a separate spreadsheet on industry.

Variables	Data Type	Content
KSIC	2-digit Integer	Korean version of the Standard Industry Code: KSIC revision 2007
Industry Code explanation	Characters	The content of the industry in the manufacturing sector
Year	4-digit Integer	From 2008 to 2014
Categories – GR	ratio	Annual Industry Growth (Industry Production_i_ – Industry Production_i-1_)/Industry Production_i-1_
Categories – CR3	%	Industry Concentration (CR3)

## 2 Experimental design, materials and methods

Korean government is keen to create a venture ecosystem in innovation cluster regions, and government agencies implement annual surveys and the target sample is all member organisations in the regions. As of December 2016, there are 5018 firms under the designated innovation clusters: 1768 in Daedeok (a region in Daejeon), 1140 in Gwangju, 728 in Daegu, 910 in Busan, and 477 in Jeonbuk. The annual survey started in Daejeon in 2006; Daegu and Gwangju joined the survey in 2011 (obtaining 2010 data). The current dataset of this article is a subset of the survey sample.

Firm selection was based on two criteria: the size of the firm and the multiple responses. Firms with fewer than 500 employees at any time during the survey period – 2008–2014 – were included. The size criteria was chosen with the aim of implementing a potential wider international comparison study in the future (e.g. definitions of manufacturing SMEs: 200 employees in the EU and 500 in the U.S.). As for the multiple responses, firms with a response frequency of five or more and exit firms with two or more were included in the dataset; this is because the associated research article [1] conducted a survival analysis demanding longitudinal data. There are five innovation clusters under the management of government agencies, but only three innovation clusters were included. The other innovation cluster regions (Busan and Jeonbuk) were excluded due to a lack of data collection before 2013, thus they do not satisfy the condition of multiple responses: five or more participation of the annual survey. Finally, as very young firms are prone to fail, firms with foundation year after 2005 were excluded.

Upon our request of technology and performance related firm questionnaire variables, government agencies of administrating three innovation cluster regions provided the coded survey results on the selected variables, where the identity of individual firm should be unnoticeable. Without special arrangement, the survey data cannot be accessed.

As for processing the dataset, we obtained the export sales and venture certificate statues of individual firms and converted them into dummy variables each year. The standard industry codes of the firms were assigned according to those obtained from the 2012 and 2008 surveys. It contains 2-digit KSIC code [3]. The growth of the industry was obtained from the Korea Statistics Office's website (KOSIS) [4] and followed a simple calculation {(Industry production_i_ – Industry Production_i-1_)/Industry Production_i-1_}. The concentration ratio of the industry was calculated from the three firm concentration ratios (CR3) that appear on Korea Development Institute (KDI)'s biannual “market structure report” under the surveillance of the government (Korea Fair Trade Commission). The report presents the associated CR3 Excel data file at 5-digit sub-industry level in recent two years, so it was possible to calculate 2-digit industry level. Although it is a biannual report, it covers the annual CR3 of the industries.

Korea's National Tax Service (NTS) compiles the closure of businesses and the information is easily accessible through the CRETOP^®^ credit information agency. The exact closure date was obtained but coded monthly in the current dataset to specify the lifespan of each firm.

**Fig. 1:**
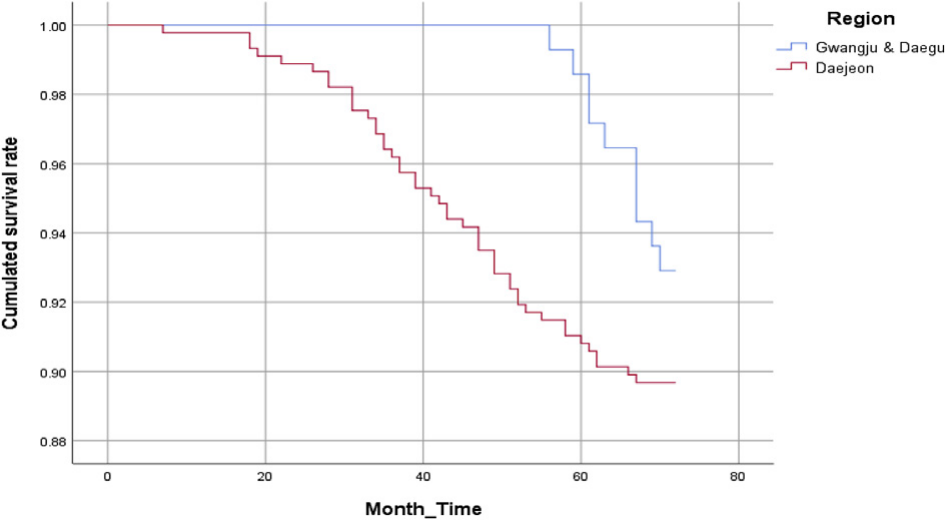
The cumulated survival function of firms in innovation clusters.

**Fig. 2:**
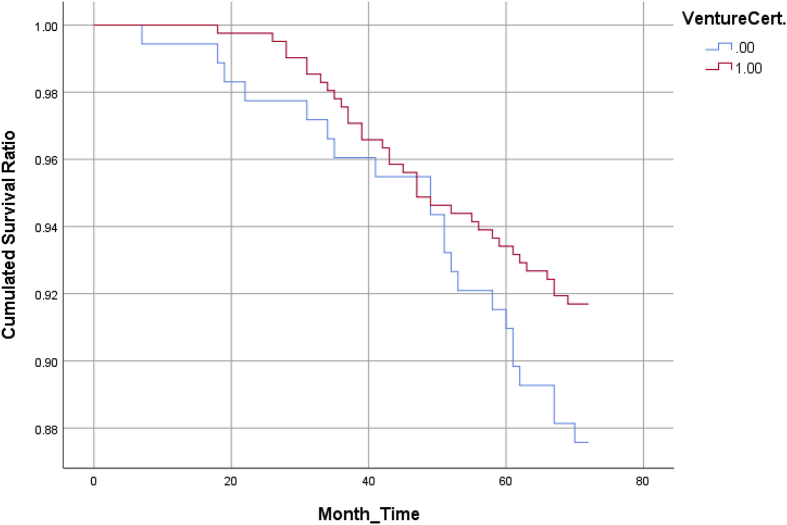
The cumulated survival function of firms with/without venture certificates.

The essential value of the data lies in the monthly records of firm survival from 2008 to 2014. Upon processing the dataset, survival patterns had been checked alongside the cumulated survival function graphs; the graphs, according to the region and firm status, are presented in Fig. 1, Fig. 2, respectively.

Basic statistics of closed (exit) firm vs. survived firm is presented in Table 4. The closed 58 SMEs are smaller and lower in technological activities, when compared with survived 530 SMEs. The dataset summarized in Table 1 was prepared for “SPSS cox regression with time varying covariates”, where time varying covariates were organized as several variables.

**Table 4:** Basic comparison of variables between closed vs. survived firms.

	Event		Mann-Whitney
	0 (closure)	1 (survived)	Sig.
	Mean	Mean	
Cases	58	550	
Num_Employee	15.82	43.66	
Sales	2232.82	17895.71	0.000
Res_Dev_Invest	171.68	3639.46	0.036
Internal_RnD_Invest	49.96	3028.26	0.000
Patent_Application	.49	1.02	0.005

The major utility of “wide format” datatset of Table 2 is as follows. After loading dataset into SPSS, a time program code, SPSS script file, similar to those used for the related research article [1], was constructed following the method of tutorial [5], and had been executed. Table 5 presents exemplary survival regression output from the SPSS script. We can observe that size (positive impact) and industry concentration (negative impact) are the most important for the SME survival amongst four variables. The script for exemplary analysis of Table 5 is also provided in a separate supplementary file (DiB_coxreg.sps in the zip file) in this data article.

**Table 5:** A Survival Analysis: Cox regression with four time varying covariates.

*Variables*	*B*	Sig.	Note
Age	-.030	0.304	Eliminated in backward stepwise cox regression
Size {Log (number of employees)}	*-.476*	0.003	The most significant explanatory variable. Minus coefficient means minus impact on hazard function, positive for survival
Number of Research Personnel	*-.060*	0.150	positive for survival, not significant.
Industry– CR3		0.001	Coefficient positive, high concentration is harmful for SME survival
−2 Log Likelyhood	*700.379*		

Note: coefficient in italics [*B*] indicate statistically significant difference between survived and closed (exit) firms.
